# SBRT in unresectable advanced pancreatic cancer: preliminary results of a mono-institutional experience

**DOI:** 10.1186/1748-717X-8-148

**Published:** 2013-06-21

**Authors:** Angelo Tozzi, Tiziana Comito, Filippo Alongi, Pierina Navarria, Cristina Iftode, Pietro Mancosu, Giacomo Reggiori, Elena Clerici, Lorenza Rimassa, Alessandro Zerbi, Antonella Fogliata, Luca Cozzi, Stefano Tomatis, Marta Scorsetti

**Affiliations:** 1 Radiotherapy and radiosurgery , Oncology, Pancreatic Surgery depts, Humanitas Cancer Center, Istituto Clinico Humanitas, Rozzano, Milano, Italy; 2 Medical Physics Unit, Oncology Institute of Southern Switzerland, Bellinzona, Switzerland; 3 Humanitas Cancer Center, Istituto Clinico Humanitas, Via Manzoni 56, 20089, Rozzano, Milano, Italy

**Keywords:** Pancreas, RapidArc, Stereotactic body radiation therapy

## Abstract

**Background:**

To assess the efficacy and safety of stereotactic body radiotherapy (SBRT) in patients with either unresectable locally advanced pancreatic adenocarcinoma or by locally recurrent disease after surgery.

**Methods:**

Between January 2010 and October 2011, 30 patients with unresectable or recurrent pancreatic adenocarcinoma underwent exclusive SBRT. Twenty-one patients (70%) presented with unresectable locally advanced disease and 9 patients (30%) showed local recurrence after surgery. No patients had metastatic disease. Gemcitabine-based chemotherapy was administered to all patients before SBRT. Prescription dose was 45Gy in 6 daily fractions of 7.5Gy. SBRT was delivered using the volumetric modulated arc therapy (VMAT) by RapidArc. Primary end-point of this study was freedom from local progression (FFLP), secondary end-points were overall survival (OS), progression free survival (PFS) and toxicity.

**Results:**

Median Clinical Target Volume (CTV) was 25.6 cm^3^ (3.2-78.8 cm^3^) and median Planning Target Volume (PTV) was 70.9 cm^3^ (20.4- 205.2 cm^3^). The prescription dose was delivered in 25 patients (83%), in 5 patients (17%) it was reduced to 36Gy in 6 fractions not to exceed the dose constraints of organs at risk (OARs). Median follow-up was 11 months (2–28 months). FFLP was 91% at 6 months, 85% at median follow-up and 77% at 1 and 2 years. For the group with prescription dose of 45Gy, FFLP was 96% at 1 and 2 years. The median PFS was 8 months. The OS was 47% at 1 year and median OS was 11 months. At the end of the follow-up, 9 patients (32%) were alive and 4 (14%) were free from progression. No patients experienced G ≥ 3 acute toxicity.

**Conclusions:**

Our preliminary results show that SBRT can obtain a satisfactory local control rate for unresectable locally advanced and recurrent pancreatic adenocarcinoma. This fractionation schedule is feasible, and no G ≥ 3 toxicity was observed. SBRT is an effective emerging technique in the multi-modality treatment of locally advanced pancreatic tumors.

## Background

Prognosis of pancreatic adenocarcinoma is still challenging, because of occult and evident metastatic disease at the time of diagnosis [1]. For those patients with no evidence of distant metastasis, multimodality approach (surgery, chemotherapy and radiotherapy) increase survival and local control rates, thus improving quality of life [2]. Only 20% of the patients, however, are considered suitable for surgery at the time of diagnosis, whereas 30-50% of the patients present with unresectable locally advanced disease [3]. In the latter group, the only therapeutic option available is the concomitant chemo-radiation treatment (CRT).

Unfortunately, despite the use of different schedules of conventional radiation and concurrent chemotherapy, local control rate after CRT is still relatively low, ranging from 40% to 55%, with a median survival ranging from 5 to 14 months [4-6].

In the last years, innovations of radiation techniques have promoted the use of hypo-fractionated regimens, allowing to improve local control for lung and liver cancer [7]. This trend has been also confirmed in other studies on stereotactic body radiotherapy (SBRT) for locally advanced pancreatic cancer, although with a higher incidence of gastrointestinal toxicity, due to the radio-sensitivity of normal organs of the upper abdomen, such as the stomach and the duodenum [8-16].

The aim of this study was to analyse the feasibility and the efficacy of hypo-fractionated SBRT in the setting of non-metastatic, unresectable primary or recurrent pancreatic adenocarcinoma.

## Methods

### Patients and eligibility

Between January 2010 and October 2011, 30 consecutive patients with unresectable or recurrent pancreatic adenocarcinoma were enrolled in this prospective, single-institutional study.

Inclusion criteria were: 1) Histologically-proven unresectable primary or recurrent pancreatic adenocarcinoma, 2) Neo-adjuvant chemotherapy 3) Age ≥18 years, 4) Karnofsky Performance score of at least 70, 5) Lesions with maximum diameter not exceeding 5 cm, 6) Ability to maintain the set-up position during RT. Exclusion criteria were: 1) Previous abdominal SBRT, 2) Metastatic disease, 3) Gastric or duodenal obstruction, 4) Concurrent chemotherapy.

### Stereotactic body radiation

Patients were immobilized in supine position with arms over the head, using a thermoplastic body mask including a styrofoam block for abdominal compression to minimize internal organ motion (spontaneous or breath-induced). A barium meal was administered to all patients about 15 mins before CT scan to enhance the stomach, the duodenum, and the small bowel. CT scan was performed with a slice thickness of 3 mm and with and without non iodinate contrast media.

The clinical target volume (CTV), defined as the gross disease, was delineated on the arterial phase of CT scan.

An additional margin of 5 mm in the left-right direction, 5 mm in the anterior-posterior direction and 10 mm in the cranial-caudal direction was added for the planning target volume (PTV). The organs at risk (OARs) including stomach, duodenum, kidneys, liver, and spinal cord, were contoured and PTV was cropped so that there was almost a 2 mm margin between the end of PTV and the start of any stomach or duodenal tissue. The dose-volume constraints for OARs were: duodenum: D_1cm3_ < 36Gy; stomach and small bowels: D_3cm3_ < 36Gy; kidneys: V_15Gy_ < 35%; liver: total spared volume (V_tot_-V_21Gy_) > 700 cm^3^; spinal cord: D_1cm3_ < 18Gy.

The prescription dose was 45Gy in 6 consecutive fractions of 7.5Gy, the required target coverage was defined as V_95%_ = 100% for the CTV. The maximum acceptable dose heterogeneity to the CTV was D_98%_ > 95% and D_2%_ < 107%. For PTV the same objectives were ideally to be achieved but with a lower priority than the constraints to the OARs. Prescription dose was reduced to 36Gy in 6 fraction of 6Gy in those cases where it was impossible to comply with dose constraints of OARs.

SBRT plans were optimized and delivered according to the volumetric modulated arc (VMAT) technique in its RapidArc form. A beam energy of 6-10MV with flattened or un-flattened (FFF) photon beams was selected for all patients. Patients were treated either on a Clinac2100 or on a TrueBeam linear accelerator (Varian Medical Systems, USA). Single or multiple coplanar partial arcs were adopted according to dosimetric requirements. Dose calculations were performed with the Anisotropic Analytical Algorithm with a grid resolution of 2.5 mm. RapidArc optimization was performed with the PROIII algorithm (Eclipse version 10).

To reduce inter-fractional positioning problems, for all patients, fasting was required at least 3 hours before the treatment session to avoid the shift of stomach.

Image guidance was performed by means of Cone beam CT imaging (CBCT) before every treatment session to verify the exact position of the patient. When necessary, couch repositioning was performed after automatic matching of CBCT images to the reference planning CT, followed by manual refining. Matching was performed on bones and, when possible, on soft tissue structures (e.g. main blood vessels).

### Response evaluation and follow-up

Patients were re-evaluated 1 month after SBRT and then every 3 months thereafter by the treating radiation oncologist. Clinical examination, CA19-9 levels evaluation and a contrast-enhanced CT were performed at each step of the follow-up. A PET-CT scan was also performed every 6 months after SRBT in those patients who had a pre-SBRT staging PET-CT scan. Local control was defined according to RECIST criteria [17] and by stable, decreasing, or normalized CA 19-9 values. Acute and late toxicity was scored according to the NCI Common Terminology Criteria for Adverse Events (CTCAE) v3.0.

### Statistical analysis

Actuarial local control and distant progression rates and overall survival were calculated from the date of SBRT to the date of progression and to the day of last follow-up or death by using the Kaplan-Meier method. All enrolled patients were included in the statistical evaluation.

## Results

### Patients and treatment characteristics

Thirty patients with pancreatic adenocarcinoma were included in this study. Twenty-one patients showed an unresectable disease invading vessels or adjacent structures while 9 patients presented with local recurrence after surgery. Patients characteristics are shown in Table 1.

**Table 1:** Summary of patient characteristics

**Patients number**	**30**
Mean age (range)	67 (43–87)
Gender (M:F)	20:10
Initial tumor characteristics	
T2	8 (27%)
T3	13 (43%)
T4	9 (30%)
N1	12 (40%)
Tumor location (number of patients):	
Head	21 (70%)
Body / Tail	9 (30%)
Mean volume (range) [cm^3^]	
CTV	25.6 (3.2-78.8)
PTV	70.9 (20.4-205.2)
Prior therapy (no. of patients)	
Surgery	9 (30%)
Chemotherapy	30 (100%)
Radiation therapy	0 (0%)

Median follow up was 11 months (range 2-28 months). Twenty-eight patients completed follow up, while two patients were unavailable and considered lost to follow up. Nine (32%) patients were alive at the time of analysis. Median follow-up was 19 months in this group of patients (range 13–28 months). The shortest follow up was due to early death of the patients. All patients received pre-SBRT gemcitabine-based chemotherapy, completed 2 weeks before SBRT at least. Ten of the patients (33%) received Gemcitabine only, 11 patients (37%) were treated with Gemcitabine-Oxaliplatinum (GEMOX), 7 patients (23%) received Gemcitabine-5-Fluorouracil (GEM-5FU) and 2 patients (7%) received Cisplatinum-Epirubicin-Fluorouracil-Gemcitabine (PEF-G). Chemotherapy cycles ranged between 3 and 14.

Forty percent of patients (n = 12) had nodal disease at time of diagnosis. Nine of these patients were treated with surgery and enrolled in this study for local recurrence of disease with no nodal involvement. In 3 patients with inoperable locally advanced cancer, a complete regression of nodal disease was achieved with pre-SBRT chemotherapy. In these 3 patients, chemotherapy was also administered after SBRT.

The radiation dose prescription was 45Gy in 6 fractions in 25 patients (83%). In 5 patients (17%) the dose prescription was reduced to 36Gy in 6 fractions not to exceed dose constraints of duodenum and stomach. Median CTV was 25.6 cm^3^ (range 3.2-78.8 cm^3^) and median PTV was 70.9 cm^3^ (range 20.4-205.2 cm^3^).

### Dosimetric features

**Figure 1:**
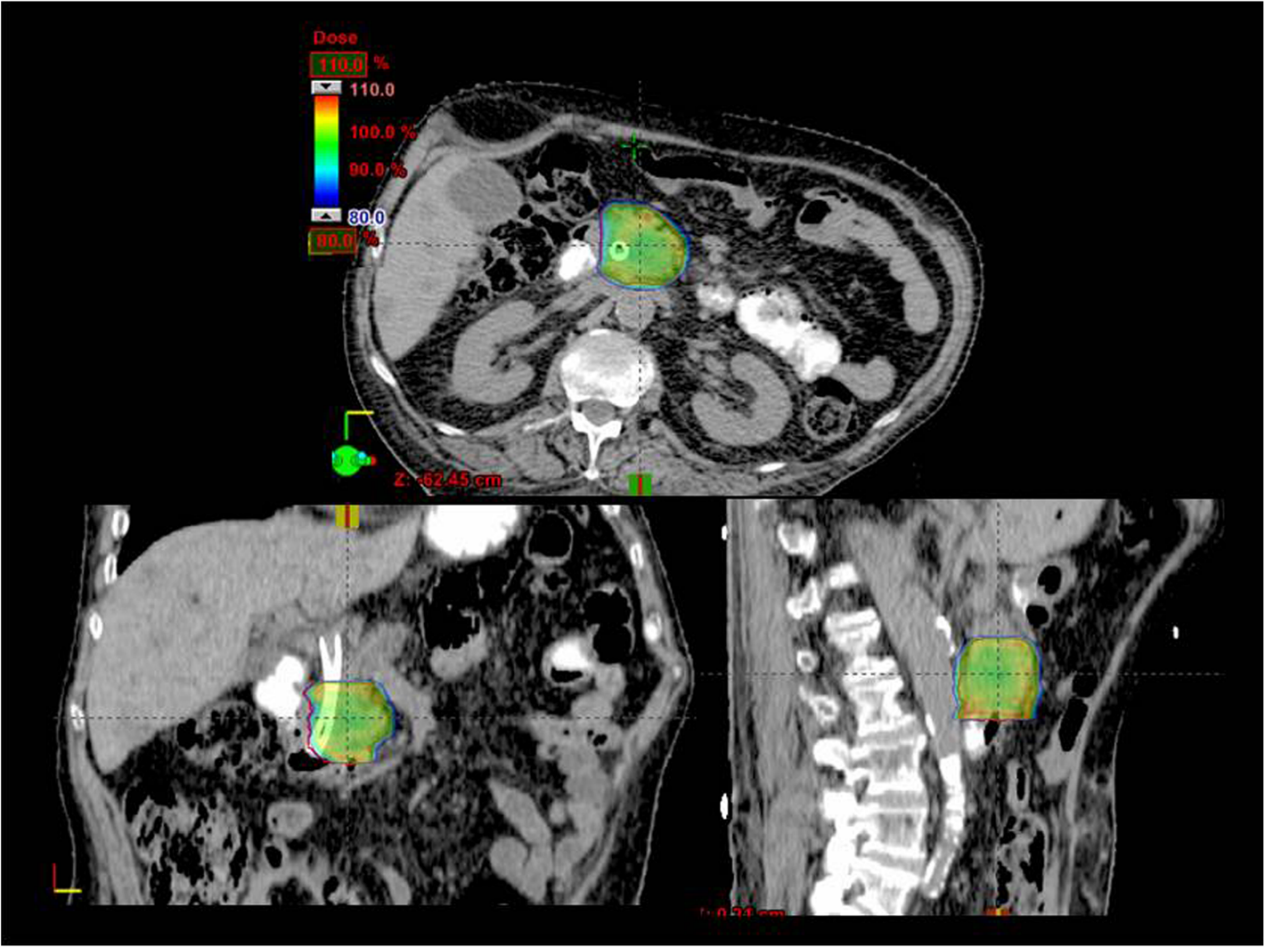
**Example of dose distribution in axial, coronal and sagittal views for a representative patient.** Color-wash thresholds are set to 80-110% (36-49.5 Gy).

**Figure 2:**
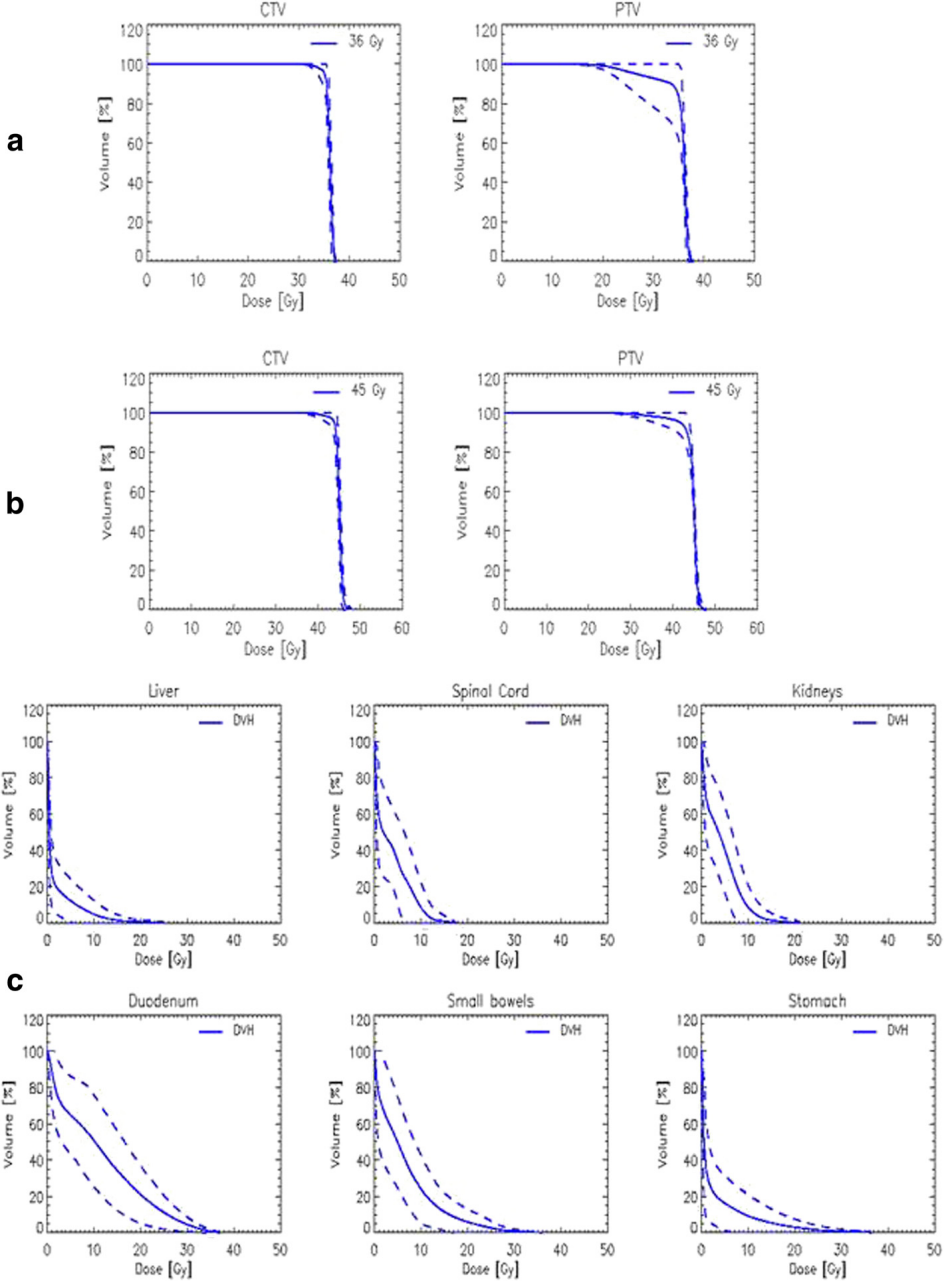
**Average cumulative DVH over the patient population (solid line), inter-patient variability is expressed at 1 standard deviation (dashed lines)**; **a**: CTV and PTV for patients treated at 36Gy; **b**: CTV and PTV for patients treated at 45Gy; **c**: OARs, irrespective of the dose prescription.

Figure 1 shows the isodose distribution in axial, sagittal and coronal views for one representative patient. Color-wash was set in the range 80%-110% (36.0-49.5Gy). Figure 2 shows the average cumulative Dose Volume histograms computed over the full patient population (a: CTV and PTV at 45Gy, b: CTV and PTV at 36Gy, c: OARs irrespective of prescription). Dashed lines correspond to the inter-patient variability expressed at 1 standard deviation. Table 2 summarizes the dosimetric characteristics of the treatment plans derived from the analysis of the DVHs. For target volume the table reports only the findings for the sub-group treated at 45Gy. Similar findings were obtained for the group treated at 36Gy but are not reported because of the limited statistics.

**Table 2:** Summary of the DVH analysis for the CTV and PTV (for the sub-group treated at 45Gy) and for the organs at risk for the entire cohort of patients

**Organ**	**Parameter**	**Objective**	**Mean ± SD**	**Range**
CTV (45Gy)	Mean [Gy]	(45Gy)	45.0 ± 0.3	[44.2;45.8]
	V_95%_ [%]	100%	98.8 ± 3.9	[95.8;100.0]
	D_98%_ [%]	>95%	95.5 ± 5.5	[93.0;102.2]
	D_2%_ [%]	<107%	104.0 ± 1.3	[100.0;106.4]
PTV (45Gy)	Mean	(45Gy)	44.5 ± 0.6	[42.9;45.1]
Left Kidney	Mean [Gy]	-	3.8 ± 1.8	[0.5;7.7]
	D_1cm3_ [Gy]	-	9.6 ± 3.6	[2.2;16.8]
	V_15Gy_ [%]	<35%	0.4 ± 1.0	[0.1;4.5]
Right Kidney	Mean [Gy]	-	4.3 ± 2.2	[0.8;10.4]
	D_1cm3_ [Gy]	-	11.8 ± 4.4	[4.4;23.8]
	V_15Gy_ [%]	<35%	1.4 ± 3.9	[0.1;17.4]
Spinal Cord	D_1cm3_ [Gy]	<18Gy	9.5 ± 3.4	[5.4;17.9]
Duodenum	D_1cm3_ [Gy]	<36Gy	29.3 ± 8.5	[9.9;36.1]
Stomach	Mean [Gy]	-	2.8 ± 2.5	[0.1;8.4]
	D_3cm3_ [Gy]	<36Gy	10.2 ± 9.8	[0.2;25.3]
Small Bowel	Mean [Gy]		6.7 ± 3.6	[0.5;14.6]
	D3_cm3_ [Gy]	<36Gy	16.1 ± 8.3	[2.3;26.5]
Liver	Mean [Gy]	-	1.8 ± 1.6	[0.3;6.0]
	V_spare_ [%]	Vtot–V21Gy > 700 cm^3^	1305 ± 290	[694;1716]

Data are reported as average values plus or minus standard deviation and range.

### Freedom from local progression (FFLP) and progression free survival (PFS )

Local control was 86% (24/28 patients). Based on CT assessment, partial response was observed in 7 patients (25%), stable disease in 17 (61%), local progression occurred in 4 patients (14%), no complete response was obtained. In 6 patients PET was available for follow up, 4 patients (67%) showed complete response and 2 patients (33%) disease local progression.

**Figure 3:**
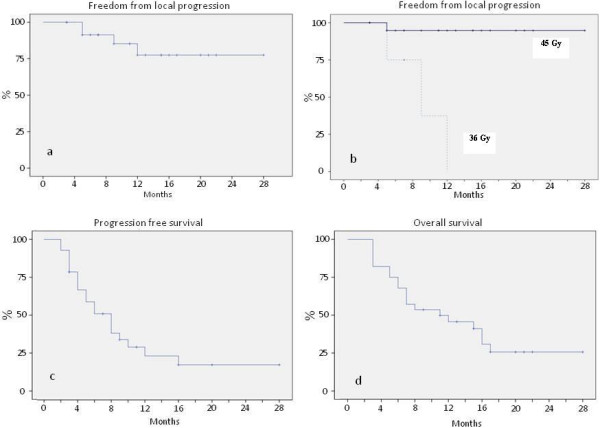
**Actuarial analysis. a**) Freedom from local progression for the entire cohort; **b**) Freedom from local progression for the patients treated with 45Gy; **c**) Time to progression; **d**) Overall survival.

Disease progressive occurred in 20 patients; progression free survival (PFS) is shown in Figure 3c. Median TTP, considered as the median duration of survival free from either local or distant progression calculated from the end of SBRT, was 8 months. Median TTP calculated from diagnosis was 14 months.

Of the 4 patients with local progression at CT assessment, 3 presented with unresectable diseases at diagnosis and 1 recurred after surgery. Time to progression was 5 months in 2 cases (1 treated with 36 Gy and 1 treated with 45 Gy), 9 and 12 months in 1 case respectively, both treated with 36 Gy. Three patients died while 1 was alive at the last follow-up.

Sixteen patients (57%) developed distant metastases. Only 4 patients were alive at the last follow up.

The FFLP rate was 93% at 6 months, 86% at median follow-up and 75% at 2 years (Figure 3a). A detailed analysis of our results showed a FFLP rate of 96% at 1 and 2 years in the group treated with a prescription dose of 45Gy with a single case of local progression (Figure 3b).

### Overall survival (OS)

The overall survival calculated from SBRT was 67% (95% CI: 0.5-0.8%) at 6 months and 47% (95% CI: 0.3-0.7%) at 1 year, with a median OS of 11 months (Figure 3d). At last follow-up, 9 patients (32%) were alive. Four patients (44%) presented with metastatic disease, 1 patient (11%) presented with local progression and 4 patients (44%) were free from progression as determined by stabilization of tumor markers with stable CT. Median follow-up in the latter group was 22 months (range 19-28 months).

### Toxicity

Twelve patients (43%) experienced fatigue 4 weeks after SBRT requiring no treatment (Grade 1). Five patients (25%) suffered from nausea G1 while additional antiemetic drugs (ondansetron) were administered to 3 (10%) patients (G2). None of these patients had persistent nausea after 1 month. Three patients (10%) presented with pain G2. There was no acute and/or late G3 toxicity. According to the Numerical Rating Scale (NRS) scoring system, 11 (37%) patients experienced pain before SBRT. In 7 patients pain control after treatment allowed suspension of analgesics administration; in 3 patients, analgesics dosage was reduced by 50%, in 1 patient administration was reduced by 20%.

## Discussion

Prognosis in patients with non-metastatic unresectable locally advanced pancreatic cancer is worsened by the inoperability judgment.

Chemotherapy alone reduces the incidence of distant metastases in patients with localized disease, with a median survival range of 9-14 months, even though it may hardly improve disease local control [18,19].

This situation leads to a detriment of the quality of life and the prognosis of this subset of patients, since local progression highly increases the risk of severe complications such as gastric and biliary obstruction, bleeding and chronic pain.

On the other hand the role of CRT in increasing the local control is controversial, because of its limited efficacy. The local progression rates reported with conventionally fractionation of RT are 40–55% [4-6].

In the last years, the unsatisfying results of conventional RT led to several studies which have investigated the feasibility and efficacy of stereotactic techniques for the treatment of pancreatic neoplasms. Recently, the encouraging results of SBRT applied to primary and secondary lesions of the lung and liver and other various sites [7,17-24], indeed, promoted several studies on hypo-fractionation technique in pancreatic cancer, as shown in Table 3. Although many of these studies were performed on a small cohort of patients whose characteristics were not always homogeneous, improvement of local control was relevant, with a success rate of 70-90% [8-16]. In the Phase II trial of Schellemberg et al, FFLP rate was 94% at 1 year and 80% at 2 years [12].

**Table 3:** Summary of treatment regimen, local control, progression free survival, overall survival and late toxicity in recent study compared to the present study

**Author, study (ref.)**	**Patients (n)**	**SBRT dose (Gy/fraction)**	**CT gemcitabina-based**	**FFLP (%)**	**PFS (months)**	**OS (months)**	**GI toxicity ( ≥ G2) (%)**
**Koong**[12]	15	15–25 Gy/1fx	no	77%	2	11 from diagnosis	none
**Hoyer**[13]	22	45 Gy/3fx	no	57%	4.8	5.7 from diagnosis	18%
**Schellenberg**[14]	16	25 Gy/1fx	sequential chemotherapy	81%	9	11.4 from diagnosis	47%
**Chang**[15]	77	25 Gy/ 1fx	For same patients prior CT	84%	-	11.4 from diagnosis	13%
**Schellenberg**[16]	20	25 Gy/1fx	sequential chemotherapy	94%	9.2	11.8 from diagnosis	20%
**Polistina**[17]	33	30 Gy/3fx	Prior chemotherapy	82.6%	7.3	10.6	none
**Didolkar**[18]	85	15–30 Gy/3 fx	sequential chemotherapy	91.7%	-	18.6 from diagnosis 8.6 from SBRT	22%
**Mahadevan**[19]	39	24-36 Gy/3fx	sequential chemotherapy	85%	15 from diagnosis	20 from diagnosis	9%
**Rwigema**[20]	71	18–25 Gy/1fx	no	64.8%	-	10.3	10%
**Present study**	30	36-45Gy/6 fx	Prior chemotherapy	85% (96% for group of 45 Gy)	8 from SBRT 14 from diagnosis	11 from SBRT 19.5 from diagnosis	none

Dx%: dose received by at least x% of the volume; Vx%: volume receiving at least x% of the dose.

If we consider PFS and OS, data were comparable to those obtained with the conventional fractionation. In the study of Mahadevan et al. and Didolkar et al, median OS was 20 and 18.6 months respectively, even though these data were calculated from the diagnosis and not from the SBRT treatment, as widely shown in other experiences [14,15].

If on one hand SBRT is effective in improving local control of unresectable pancreatic cancer, on the other hand acute and late toxicity are still challenging. The rate of late gastro-duodenal toxicity G2 or greater varies from 10% and 50% in several studies.

In our study all patients were treated for unresectable locally advanced pancreatic adenocarcinoma, with a prescription dose of 45Gy in 6 fractions in 85% of patients. In 5 patients, however, prescription dose was reduced to 36Gy in 6 fractions, as not to exceed dose constraints at duodenum, stomach and small bowel. We reported the FFLP rate of 85% at median follow-up and 76% at 2 years. Particularly, in the group of 25 patients treated with 45Gy, FFLP was 96%, at 1 and 2 years, confirming the efficacy of this fractionation, especially when there is no need of a dose reduction.

Moreover there was no difference in the local control between the patients treated with SBRT at diagnosis and those treated for local recurrence after surgery, since only 1 patient of the latter group presented with local progression.

Another key-point of this study is the gemcitabine-based chemotherapy before SBRT, although there is no homogeneity of the adopted schedules. This therapeutic strategy aims to control undetected distant micrometastases at the time of diagnosis and treatment. Metastatic disease is the usual way of progression and also in our experience 80% of the 20 patients with progressive disease, presented with distant metastases. The median TTP calculated from the start of SBRT and from the diagnosis was 8 months and 11 months, respectively. The median OS calculated from the start of the treatment was 14 months, comparable to those results reported by several studies in the literature [14,15].

Even if better local control does not improve significantly the survival, it may reduce the risk of gastric and biliary obstruction and other morbidities. None of the patients free from local progression in this study, indeed, showed any of these serious complications.

Several previous experiences demonstrated that a hypo-fractionated approach is more effective to treat small size tumors. In our study, however, larger volumes were treated with an optimal outcome, also with regard to toxicity. For pancreatic RT, the presence of near dose-limiting structures can be considered a limit. Stereotactic regimens are associated with an incidence of G ≥ 3 acute toxicity lower than with conventionally fractionated radiation therapy [4-6], but the late risk of ulcers and bleeding continues to be significant with an incidence of 10-47% [10-12,14]. The biologically equivalent dose of our regimen is superior to conventionally fractionated external beam radiation, both for tumor and normal tissue toxicity, but within the limits of tolerance for the gastrointestinal tract. Unlike most of the other studies, our immobilization system with thermoplastic body mask including a Styrofoam block for abdominal compression, minimize the internal organ motion, reducing the dose to OARs. We prioritized the duodenal and stomach dose constraints so that the maximum dose of 36Gy was not exceeded (Duodenum: D1% < 36Gy; stomach: D3% < 36Gy). In 5 patients, this was not possible because of the close contiguity of PTV and the duodenum or the stomach and dose prescription was reduced to 36Gy in 6 fractions. None of our patients experienced perforation, ulcer, bleeding or other types of acute or late toxicity G ≥ 3, confirming the safety of this stereotactic body regimen. We feel that the high conformity of dose with hotspots smaller than those reported in other papers, allowed us to achieved this optimal toxicity profile. Also, in all patients with pain at the time of SBRT we obtained a reduction of the symptoms, according to those reported in literature [8-16].

In conclusion, within the limitations of a relatively small sample size, this study demonstrated that SBRT is an efficacy and safety therapeutic option to improve local control in patients with unresectable advanced pancreatic adenocarcinoma. Future studies are needed to identify the best therapeutic strategy in combination with systemic therapy to increase the impact on survival for these patients.

## Competing interests

Dr. L. Cozzi acts as Scientific Advisor to Varian Medical Systems and is Head of Research and Technological Development to Oncology Institute of Southern Switzerland, IOSI, Bellinzona.

## Authors’ contributions

MS, FA, TC, AT, LC designed the study and the analysis. PN, CI, EC, LR, AZ, MS, AT, TC, FA collected the clinical data, PM, GR, ST collected the dosimetric data. AT, TC, ST, LC, AFC performed main data analysis. AT, TC, FA, LC drafted the manuscript. All authors reviewed and approved the final manuscript.
